# Unified Channel Management for Cognitive Radio Sensor Networks Aided Internet of Things

**DOI:** 10.3390/s18082665

**Published:** 2018-08-14

**Authors:** Saleem Aslam, Ansar-ul-Haq, Ju Wook Jang, Kyung-Geun Lee

**Affiliations:** 1 Department of Electrical Engineering, Bahria University, Islamabad 44000, Pakistan; saleem.aslam@bui.edu.pk (S.A.); engr.auhaq@gmail.com (A.-u.-H.); 2 Department of Electronics Engineering, Sogang University, Seoul 04107, Korea; 3 Department of Information and Communication Engineering, Sejong University, Seoul 143-747, Korea; kglee@sejong.ac.kr

**Keywords:** internet of things, cognitive radio, channel scheduling, quality of service, cognitive center, 5G user, IoT devices, multi-channel, square law combining, channel ordering

## Abstract

Cognitive capabilities are indispensable for the Internet of Things (IoT) not only to equip them with learning, thinking, and decision-making capabilities but also to cater to their unprecedented huge spectrum requirements due to their gigantic numbers and heterogeneity. Therefore, in this paper, a novel unified channel management framework (CMF) is introduced for cognitive radio sensor networks (CRSNs), which comprises an (1) opportunity detector (ODR), (2) opportunity scheduler (OSR), and (3) opportunity ranker (ORR) to specifically address the immense and diverse spectrum requirements of CRSN-aided IoT. The unified CMF is unique for its type as it covers all three angles of spectrum management. The ODR is a double threshold based multichannel spectrum sensor that allows an IoT device to concurrently sense multiple channels to maximize spectrum opportunities. OSR is an integer linear programming (ILP) based channel allocation mechanism that assigns channels to heterogeneous IoT devices based on their minimal quality of service (QoS) requirements. ORR collects feedback from IoT devices about their transmission experience and generates special channel-sensing order (CSO) for each IoT device based on the data rate and idle-time probabilities. The simulation results demonstrate that the proposed CMF outperforms the existing ones in terms of collision probability, detection probability, blocking probability, idle-time probability, and data rate.

## 1. Introduction

Internet of things (IoT) is the promising solution to address the challenges of smart transportation, smart governance, smart industry, smart management of daily life matters and so on [1,2]. However, due to their unprecedented numbers, which are expected to reach 30 billion by 2020, it is quite challenging to meet their expectations in terms of spectrum, coverage, and data rate [3]. Furthermore, existing technologies for IoT (e.g., RFID, UWB, Zigbee, etc.), due to their shorter range, results in the formation of small isolated networks [4,5]. These large numbers of isolated and disconnected networks create one of the big hurdles in the realization of the larger picture of IoT such as smart cities and smart environments. To address the above challenges and constraints, the wide-band technologies can be a viable solution. A cognitive radio sensor networks (CRSN) is one of the key candidates in this regard, which not only provides a wide range of non contiguous spectrum but also manages to meet the stringent quality of service (QoS) requirements of diverse IoT devices [5,6].

- Opportunity Detector (ODR): An ODR scheme allows the user of secondary tier to opportunistically access the vacant portion of the spectrum by sensing the activity of the primary tier user (PTU) [7,8,9]. There are numerous ways to perform ODR including matched filtering, cyclo-stationary feature detection and energy detection. However, the energy detection (ED) is the most commonly used technique because of its low computational complexity and ease of implementation [10]. Urkowitz et al. [11] derive the formula for calculating the probability of detection (PoD) and probability of false alarms (PoFA) for AWGN channels. ED can be implemented in two different ways: (1) with a single threshold and (2) with the double threshold. In the former mechanism, the ODR compares the detected energy to a single threshold, whereas the detected energy is compared to two predefined thresholds in the later mechanism [12]. Ahmadian et al. [13] present a single threshold based ED scheme. The authors analyze the PoD over different fading channels. Their scheme results in a higher PoD, which in turn gives better protection for PTU. However, it also gives a higher PoFA, which shows fewer chances of channel utilization for secondary tier users (STUs) even when the channel is available. To minimize false alarms, a double threshold based ED mechanism is introduced in [14,15,16,17] where the authors minimize the PoFA and probability of collision (PoC). In these schemes, the signals received from the PTUs are simply amplified and forwarded to the cognitive center where different combining techniques are used such as maximal combining ratio (MRC), square law combining (SLC) and equal gain combining (EGC). In [18,19,20,21,22,23,24], the authors analyze the performance parameters of PoC, PoD, PoFA, energy, and secondary user (SU) utility over AWGN and Nakagami-m fading channels with the claim of improving all aforementioned parameters. However, the scope of all these schemes is limited to the detection of a single channel, which limits their spectral usage and scope for densely (i.e., areas where a large number of devices operate in the close vicinity) populated environment.
  Few efforts have been made in the domain of multichannel sensing for opportunistic networks [25,26,27]. Although authors claim improvement in the probability of detection and probability of false alarms, the system models of these schemes rely on a single threshold mechanism, which limits their effectiveness across the collision probability. To the best of our knowledge, there is no literature available that incorporates a sophisticated cooperative mechanism for the multichannel environment. Furthermore, the double threshold mechanism is also not explored in the multichannel ODR. Therefore, in this paper, we merge all objectives to develop a robust ODR mechanism based on a multichannel cooperative spectrum-sensing scheme with a double threshold mechanism to cater to the limitations of existing schemes and to improve detection probability, reliability and throughput.
- Opportunity Scheduler (OSR): The OSR scheme allocates the channel to secondary tier devices for their transmission. For opportunistic access of spectrum, one of the key elements is the stability of the channel. By stability, we mean how smoothly IoT devices conduct communication during frame *f* without interruption from the 5G user. The authors of [28,29] introduce a dynamic channel selection scheme (DCSS) and dynamic random channel selection scheme (DRCSS) to optimize the throughput. Several OSR schemes [30,31,32] have been proposed in the literature for QoS provisioning. Although the authors show performance gain in terms of throughput, all of these schemes are centric towards the cellular environment. In [33], the authors present a scalable channel allocation scheme for IoT. The performance is analyzed in terms of fairness and throughput, which lacks the stability analysis of the channel. The real-time measurement based channel prediction and allocation scheme are presented in [34], where performance gain is shown in terms of throughput and power. However, the scheme is highly dependent on the location of the user, which limits its use in the mobile environment. Furthermore, the activity of the PTUs is not considered in problem formulation. The authors in [35] present an OSR scheme that considers QoS parameters and activity of the PTU. However, this scheme does not satisfy the data rate requirement of different applications running on IoT devices. Similarly, the OSR mechanisms introduced in [36,37,38] ensure energy efficiency but lack consideration of the presences of the PTU and device level QoS of IoT devices. In a similar manner, the authors in [39,40] focused only on minimizing the congestion at the base station of the secondary tier and do not provide device level QoS. Therefore, we introduce a robust OSR scheme that considers both quality and stability of channels to meet the minimal QoS of the IoT devices.
- Opportunity Ranker (ORR): The role of ORR is to collect the feedback from IoT devices during frame *f* and prepare channel sensing order (CSO) for frame *f*+1. A robust ORR can significantly enhance the performance of ODR and OSR by allowing IoT devices to consider high quality and stable channels during ODR and ORR processes.

### Contributions

To the best of our knowledge, the feedback based ORR is the first of its kind that collects real-time feedback from IoT. Furthermore, the existing literature on channel sensing and channel scheduling do not address following cases: (1) coexistence model of 5G and IoT in a two-tier format, (2) ODR based on multichannel spectrum sensing with double threshold mechanism, and (3) channel allocation considering device level QoS and stability of the channel. The main contributions of this paper can be summarized as follows:

1. A new perspective of two-tier communication is introduced for the coexistence of 5G and IoTs. Modeling of the proposed two-tier network is done by employing CR techniques where 5G communication belongs to primary tier and IoT operates at the secondary tier. The IoT devices opportunistically access the spectrum band of 5G users.
2. A novel unified CMF is introduced which is comprised of ODR, OSR and ORR. ODR modeling in a multichannel environment is the first of its kind, which enables IoT devices to sense multiple channels concurrently to locate more opportunities. Furthermore, ODR is equipped with a double threshold classifier and combining diversity mechanisms to further reduce the PoC and improve detection performance.
3. To fulfill the QoS requirements of heterogeneous IoT devices, a novel integer linear programming (ILP) based OSR scheme is proposed which allocates the best channels to IoT devices to meet their minimal data rate requirements considering channel’s quality and stability.
4. To improve the performance of ODR and OSR, a novel ORR scheme is introduced which collects channel feedback in terms of data rate and 5G user arrival activity from IoT devices and prepares a special CSO for each IoT device *i*. This CSO lists only those channels that are best in terms of quality and stability.

The rest of the paper is organized as follows. Section 2 describes the system model (i.e., network model, frame format, IoT traffic types, etc.) and existing ODR mechanisms. Section 3 describes ODR, OSR, and ORR schemes of the proposed CMF. Simulation results are presented in Section 4. Finally, Section 5 concludes the outcomes.

## 2. System Model

### 2.1. Network Model

**Figure 1:**
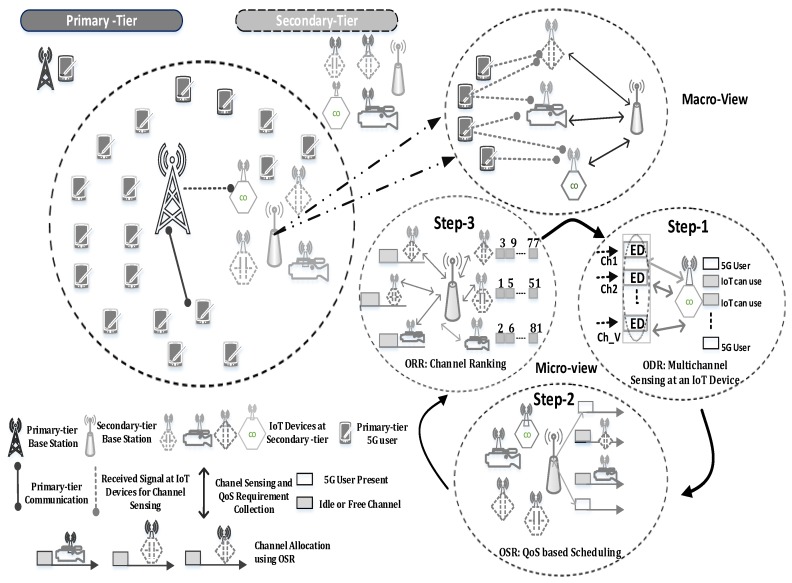
A novel unified channel management framework for the coexistence of IoT and 5G communication.

A two-tier network is considered where S number of 5G users (i.e., PTUs) and N IoT devices (i.e., STUs) are operating in a microcell. IoT devices can sense and access ℳ free channels (i.e., not utilized by the 5G user at a given time instant) in an opportunistic manner by employing CR techniques. The secondary tier base station (STBS) performs (1) ODR, (2) OSR, and (3) ORR functions as shown in Figure 1. In ODR, each IoT device *i* performs multichannel ED on given ℳ*^o^*⊆ℳ channels and report their results to STBS, which applies combining diversity mechanism to make the final decision about the availability (i.e., idle or busy channel) of *k*-th channel. In OSR, the STBS allocates channels based on the traffic class of the IoT devices. Each class has different QoS requirements in terms of minimum data rate $\zeta_{i}^{min}$ and idle time $\rho^{min}$. We introduce four different traffic classes such as real-time high throughput (RTHT), non real-time high throughput (nRTHT), real-time short message (RTSM), and non real-time short message (nRTSM). After acquiring channels from the STBS, the IoT devices perform data communication tasks. when IoT devices complete their transmission during frame *f*, they report their experience about data rate and 5G user activity to STBS which then forms a CSO and forwards it to each IoT device *i* for ODR during frame *f*+1. This helps the IoT devices to sense better quality channels to optimize the functionality of ODR and OSR. Without loss of generality, we assume that IoT devices, STBS and PTBS in different micro cells are perfectly synchronized and they can coordinate wherever required via predefined control channels for better coordination and interference handling. The proposed scheme is highly scalable and its scope can be extended by increasing the base station in the second tier of the network. Table 1 describes the popular terms and notations used in the rest of the paper. The terms IoT device, STUs and CR compliant devices are used interchangeably.

### 2.2. Frame Format

**Figure 2:**
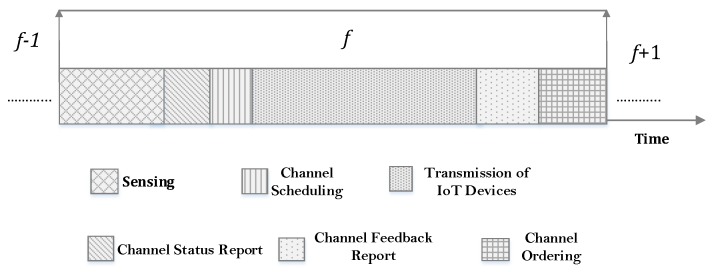
Frame format.

Figure 2 illustrates the frame format used in CMF and highlights different operations within a frame *f*. A slotted architecture is considered and it is assumed that IoT devices and STBS are perfectly synchronized. We consider that the IoT device can perform multi-channel ED over ℳ*^o^*⊆ℳ channels, where the value of $M^{o}$ is different for each IoT device, in a predefined order. To exploit the heterogeneity of IoT devices, we consider that each IoT device can sense a different number of channels based on their processing capabilities. IoT devices monitor the environment and report their observation to STBS, which directs it to the sink node via an internet. Initially, each IoT device sense $M^{o}$ channels and report their channel status report to STBS. The STBS uses the square law combining technique (SLC) to make the final decision about the availability (i.e., 5G user is absent) of a channel for IoT devices.

After that, the STBS performs an OSR task and schedules channels to IoT devices depending on their minimal QoS requirements. Once the IoT devices utilize the channel and complete their data transmission during frame *f*, they report their observations about channel condition to STBS in terms of data rate and 5G user activity. After getting channel feedback reports (CFRs) from active IoT devices (i.e., by active devices, we mean those IoT devices that perform the data communication during frame *f*), the STBS prepares a special CSO for each IoT device *i* for next frame *f*+1 to target only the stable and high quality channels.

### 2.3. Channel-Sensing

In channel-sensing, the main aim is to distinguish between the following two hypotheses [41]:

(1) $$\begin{array}{c} H^{0}:r\left(t\right)=\eta \left(t\right),\\ H^{1}:r\left(t\right)=x_{o}\left(t\right)h\left(t\right)+\eta \left(t\right), \end{array}$$

where *r*(*t*) is the received signal at IoT device *i*, $x_{o}\left(t\right)$ represents the signal of the 5G user, *h*(*t*) is the channel response and η(t) indicates the noise contents. The IoT device utilizes its spectrum sensing circuit to decide $H^{0}$ if the *k*-th channel is unoccupied, and it declares $H^{1}$ if it is occupied by a PTU. Based on the detected energy, the IoT device experiences the following distributions [42]:

(2) $$Z=\left\{\begin{array}{cc} R_{2TW}^{2}, & if\;H^{0},\\ R_{2TW}^{2}\left(2\gamma \right), & if\;H^{1}, \end{array}\right.$$

where *Z* and γ indicates the energy values sensed by an IoT device and signal-to-noise ratio (SNR) of the channel, respectively. The $R_{2TW}^{2}$ and $R_{2TW}^{2}\left(2\gamma \right)$ indicate the central and non-central chi-square distributions. The decision is made based on a likelihood ratio test, which can be described as follows [43]:

(3) $$T\left(r\right){≷}_{H^{0}}^{H^{1}}\lambda_{0},$$

where *T*(*r*) represents the test statistic of the *k*-th channel, and $\lambda_{0}$ is the threshold that divides the decision region into $H^{0}$ and $H^{1}$. For non-fading environment with deterministic *h*(*t*), the probabilities of detection $P_{d}$, collision $P_{c}$ and false alarms $P_{fa}$ are given as follows [42,43]:

(4) $$P_{d}=P\left\{Z>\lambda_{0}/H_{1}\right\}=Q_{u}\left(\sqrt{2\gamma },\sqrt{\lambda_{0}}\right),$$

(5) $$P_{c}=P\left\{Z\leq \lambda_{0}/H_{1}\right\}=1-P_{d},$$

(6) $$P_{fa}=P\left\{Z>\lambda_{0}/H_{0}\right\}=\frac{\Gamma (u,\lambda_{0}/2)}{\Gamma (u)},$$

where Γ(u) and $\Gamma (u,\lambda_{0}/2)$ represent complete and incomplete gamma functions and $Q_{u}\left(\sqrt{2\gamma },\sqrt{\lambda_{0}}\right)$ is the generalized Marcum functions [44]. The collision probability indicates the possibility of interference to PTU. Contrary to this, a high value of $P_{fa}$ indicates the possibility that an STU *i* declares the presence of PTU when it is actually absent in the channel. This will result in the lower utilization of spectrum.

To handle real environments with multi-path fading, shadowing and hidden node issues, the cooperative sensing mechanism is recommended where multiple receivers take part in the collective decision at the fusion center [41]. For real environments, the cooperative probabilities of detection $(Q_{d}$), collisions $(Q_{d}$) and false alarms $\left(Q_{fa}\right)$ can be described as follows [45]:

(7) $$Q_{d}=1-\Pi_{i=1}^{N}\left(1-P_{d}\right),$$

(8) $$Q_{c}=1-\Pi_{i=1}^{N}\left(P_{c}\right),$$

(9) $$Q_{fa}=1-\Pi_{i=1}^{N}\left(1-P_{fa}\right).$$

### 2.4. Estimated Size of Data Transmission and 5G User Activity

Let $D_{i,f,k}^{est}$ represent the estimated number of bits that an IoT device *i* can transmit on channel *k* during frame *f*, which can be calculated as follows:

(10) $$D_{i,f,k}^{est}=W_{k}log_{2}\left(1+SNR_{i,f,k}\right)T_{f}^{tx},$$

where $T_{f}^{tx}$ is the transmission time during frame *f* and $W_{k}$ is the bandwidth of the channel. For simplicity, we assume the same transmission time for all frames. The 5G user behavior can be modeled using the probabilistic model as follows [46]:

(11) $$P_{idle}^{k}\left\{t_{o}+T_{sens}|t_{o}\right\}=\frac{1-\int_{o}^{t_{o}+T_{sens}}X_{idle}^{k}\cdot dt}{1-\int_{o}^{t_{o}}X_{idle}^{k}\cdot dt},$$

(12) $$P_{busy}^{k}\left\{t_{o}+T_{sens}|t_{o}\right\}=\frac{1-\int_{o}^{t_{o}+T_{sens}}X_{busy}^{k}\cdot dt}{1-\int_{o}^{t_{o}}X_{busy}^{k}\cdot dt},$$

where *k* is the channel index, $t_{o}$ is the duration of idle/busy periods and $T_{sens}$ is the sensing times. $X^{k}$ is the probability function of idle/busy duration of the channel *k*. The higher value of $P_{idle}^{k}$ indicates higher opportunity for IoT devices to transmit over channel *k*.

### 2.5. IoT Traffic Types

Four heterogeneous traffic types are considered for IoT devices such as (1) real-time short messages (RTSM), (2) non real-time short messages (nRTSM), (3) real-time high throughput (RTHT) and (4) non real-time high throughput (nRTHT). The minimal QoS requirements are given in Table 2 [7,30].

Temperature and pressure monitoring IoT are the examples of nRTSM traffic class whereas the smoke detector is the member of RTSM class. The IoT devices providing the real-time video of a particular region of interest belong to the class of RTHT.

## 3. Proposed Novel Unified Channel Management Framework

This section provides the detailed workings of the proposed novel CMF and problem formulation of associated ODR, OSR and ORR schemes, which jointly provide the best possible channels to IoT devices for their opportunistic data transmission.

### 3.1. Opportunity Detector (ODR)

**Figure 3:**
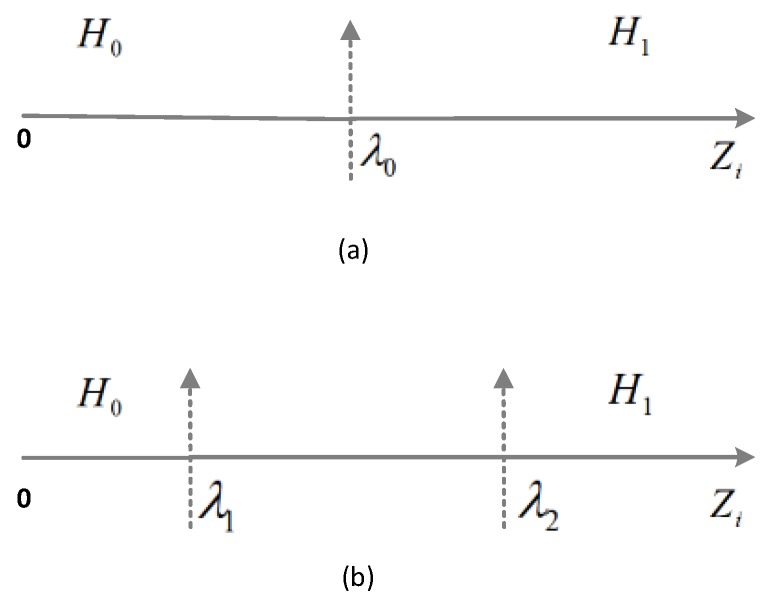
Threshold mechanisms (**a**) single threshold; (**b**) double threshold.

This subsection illustrates the mathematical modeling and working of the proposed double threshold based cooperative multi-channel ODR scheme. First, the double threshold mechanism is described and then the mathematical expression for PoD, PoC, PoFA are presented in the context of multichannel ODR. Figure 3 depicts the difference between single and double threshold mechanisms. Traditionally, the users of the secondary tier make the local decision by comparing its received energy with a predefined single threshold value $\lambda_{0}$ as shown in Figure 3a. The authors of [45] depict the double threshold energy mechanism in a single channel-sensing scenario as shown in Figure 3b. There are two thresholds $\lambda_{1}$ and $\lambda_{2}$. If detected energy $Z_{i}$ of an IoT device *i* is greater than $\lambda_{2}$, then the IoT device reports $H^{1}$ to STBS, which means that the IoT device detected 5G user activity on channel *k*. If the value of $Z_{i}$ is less than $\lambda_{1}$, the IoT device will report $H^{0}$ to STBS, indicating that the channel is free. The proposed ODR scheme can be described as follows:

In ODR, each IoT device *i* performs a spectrum sensing task over *k* = 1, 2, … $M^{o}$ channels where $M^{o}$ is recommended by the STBS as an output of ORR in the form of special CSO (see Section 3.3 for details):

(13) $$\begin{array}{c} H_{i,k}^{0}:r_{i,k}\left(t\right)=\eta_{k}\left(t\right),\\ H_{i,k}^{1}:r_{i,k}\left(t\right)=x_{k}^{o}\left(t\right)\cdot h_{k}\left(t\right)+\eta_{k}\left(t\right), \end{array}$$

where $r_{i,k}\left(t\right)$ represents the received signal of the IoT device *i* on channel *k*, $x_{k}^{o}\left(t\right)$ is the signal of the 5G user over channel *k*, $h_{k}\left(t\right)$ is the channel response and $\eta_{k}\left(t\right)$ indicates the noise contents of channel *k*. The IoT device *i* decides $H_{i,k}^{0}$ if the *k*-th channel is unoccupied, and it decides $H_{i,k}^{0}$ if it is occupied by the 5G user. Based on the detected energy, the IoT device *i* experiences the following distributions [45]:

(14) $${\bar{Z}}_{i,k}=\left\{\begin{array}{cc} Z_{i,k}, & if\;\lambda_{1}<Z_{i,k}<\lambda_{2},\\ V_{i,k}, & otherwise, \end{array}\right.$$

(15) $$V_{i,k}=\left\{\begin{array}{cc} 0, & if\;\lambda_{1}<Z_{i,k}<\lambda_{2},\\ 1, & Z_{i,k}>\lambda_{2}, \end{array}\right.$$

where ${\bar{Z}}_{i,k}$ is the information received by the STBS from IoT device *i* on channel *k*. For simplicity, we assume the same threshold values for all IoT devices. If the value of $Z_{i,k}$ lies between $\lambda_{1}$ and $\lambda_{2}$, the IoT device *i* reports actual value of $Z_{i,k}$ to STBS; otherwise, it forwards its local decision $V_{i,k}$ to STBS. We assume that STBS receives $N^{o}\subseteq N$ local decisions $V_{i,k}$, which is given as follows:

(16) $${\bar{T}}_{i,k}=\left\{\begin{array}{cc} 0, & if\;0\leq \sum_{i=1}^{N-N^{o}}\;Z_{i,k}\leq \lambda_{stbs},\\ 1, & \sum_{i=1}^{N-N^{o}}\;Z_{i,k}>\lambda_{stbs}, \end{array}\right.$$

where $\lambda_{stbs}$ is the energy detection threshold used by the STBS. To accommodate the energy values between $\lambda_{1}$ and $\lambda_{2}$, the STBS makes the final decision $\bar{D_{i,k}}$ by exploiting the combining diversity technique as follows [46,47,48]:

(17) $$\bar{D_{i,k}}=\left\{\begin{array}{cc} 1, & D+\Sigma_{i=1}^{N^{o}}V_{i,k}>1,\\ 0, & otherwise. \end{array}\right.$$

#### Performance Analysis of ODR

The performance of the proposed ODR scheme can be analyzed with the analytical expressions of ${\bar{P}}_{d,i,k}$, ${\bar{P}}_{c,i,k}$ and ${\bar{P}}_{fa,i,k}$. To make analysis simple, we define the probability for undefined region between the threshold $\lambda_{1}<V_{i,k}<\lambda_{2}$ for IoT device *i* over channel *k* under hypothesis $H_{0}$ and $H_{1}$ as follows [48]:

(18) $${\bar{H}}_{i,k}^{0}=P\left\{\lambda_{1}<V_{i,k}\leq \lambda_{2}/H^{0}\right\},$$

(19) $${\bar{H}}_{i,k}^{1}=P\left\{\lambda_{1}<V_{i,k}\leq \lambda_{2}/H^{1}\right\},$$

(20) $${\bar{P}}_{d,i,k}=P\left\{V_{i,k}>\lambda_{2}/H^{1}\right\}=Q_{u}\left(\sqrt{2\gamma },\sqrt{\lambda_{2}}\right),$$

(21) $${\bar{P}}_{c,i,k}=P\left\{V_{i,k}\leq \lambda_{1}/H^{1}\right\}=1-{\bar{H}}_{i,k}^{1}-P_{d,i,k},$$

(22) $${\bar{P}}_{fa,i,k}=P\left\{V_{i,k}>\lambda_{2}/H^{0}\right\}=\frac{\Gamma (u,\lambda_{2}/2)}{\Gamma (u)}.$$

Therefore, the cooperative probabilities of detection ${\bar{Q}}_{d,i,k}$, collisions ${\bar{Q}}_{c,i,k}$ and false alarms ${\bar{Q}}_{fa,i,k}$ for multichannel-sensing environment can be derived as follows:

(23) $$\begin{array}{c} {\bar{Q}}_{c,i,k}=\Sigma_{K=0}^{N-1}\left({}_{N^{o}}^{N}\right)\Pi_{i=1}^{N^{o}}\;P_{c,i,k}\;\Pi_{i=N^{o}+1}^{N}H_{i,k}^{1}\\ \left[1-Q_{(N-N^{o})u}\left(\sqrt{2\gamma_{0}},\sqrt{\lambda }\right)\right]+\Pi_{i=1}^{N}P_{c,i,k}, \end{array}$$

(24) $$\begin{array}{c} {\bar{Q}}_{fa,i,k}=1-\Pi_{i=1}^{N}\left(1-H_{i,k}^{o}-P_{f,i,k}\right)\\ =-\Sigma_{N^{o}=0}^{N-1}\left({}_{N^{o}}^{N}\right)\Pi_{i=1}^{N^{o}}\;\left(1-H_{i,k}^{o}-P_{f,i,k}\right)\Pi_{i=N^{o}+1}^{N}H_{i,k}^{o}\\ \left[1-\frac{\Gamma [\left(N-N^{o}\right)u,\lambda /2]}{\Gamma [\left(N-N^{o}\right)u]}\right], \end{array}$$

(25) $${\bar{Q}}_{d,i,k}=1-{\bar{Q}}_{c,i,k}.$$

These probabilities quantify the performance of the ODR using double thresholds and multichannel sensing capabilities.

### 3.2. Opportunity Scheduler (OSR)

Before Scheduling, STBS gets requirements from IoT devices in terms of their minimum data rate $\zeta_{i}^{min}$ and $\rho_{i}^{min}$ based on their classes. Since IoT devices are heterogeneous in terms of their capabilities, they need the different type of channels. For example, an IoT device generates traffic of RTHT having a different minimum data rate $\zeta_{RTHT}^{min}$ than IoT devices belonging to nRTSM $\zeta_{nRTSM}^{min}$ class. To ensure the stability analysis, we incorporate the idle time probabilities in the scheduling decision. The optimization problem of the proposed OSR scheme is described in Equation (26) where the binary variable $X_{i,k}$ indicates the allocation of channel *k* to an IoT device *i*. The solution to integer linear program (ILP) is obtained using an ’intlinprog’, function of MATLAB R2017a. The first constraint ensures the requirement satisfaction of heterogeneous IoT devices. The stability of channels is ensured using Constraint-2. The Constraint-3 and Constraint-4 quantify that only one IoT device will use one channel and at most one channel is allocated to an IoT device:

(26) $$\begin{array}{cc} \mathrm{Maximize} & \sum_{i=1}^{N}\sum_{k=1}^{M}D_{i,f,k}^{est}X_{i,k},\\ \mathrm{Subject}\;\mathrm{to} & D_{i,f,k}^{est}\geq \zeta_{i}^{min}\;\forall i,k,\\ & P_{idle}^{k}\geq \rho_{i}^{min}\;\forall i,k,\\ & \sum_{i=0}^{N}X_{i,k}\leq 1,\\ & \sum_{k=0}^{M}X_{i,k}\leq 1,\\ & X_{i,k}=\left\{0,1\right\}. \end{array}$$

### 3.3. Opportunity Ranker (ORR)

**Figure 4:**
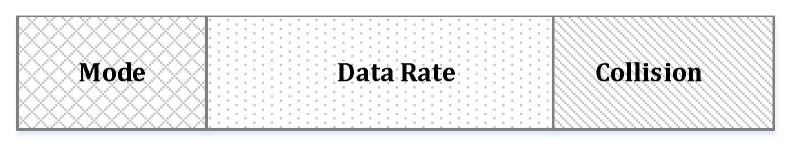
Channel feedback report.

After completing the transmission over channel *k* acquired through OSR scheme, each IoT device *i* reports their feedback to STBS about their real experience of transmission during frame *f*. The STBSS performs an ORR algorithm to prepare special CSO for the IoT devices *i* for next frame *f*+1. The IoT devices report their experience to STBS using the channel feedback reports (CFRs) that contain node ID, data rate and collision fields as shown in Figure 4. The node ID is the identifier of IoT device *i*. The data rate field contains the actual data rate achieved by the IoT device in frame *f*. The collision field indicates the arrival of 5G user on the same channel that is allocated by the STBS to an IoT device *i* using OSR.

The STBS calculates the average data rate and average 5G user activity using CFRs and applies the TOPSIS method [49] to prepare CSO for each IoT device *i* for frame *f*+1. The TOPSIS method ranks the channels by exploiting similarity and dissimilarity to the ideal solution by reducing the distance to the ideal positive solution and increasing the distance from the ideal negative solution. The TOPSIS based modeling of ORR is as follows [50].

Let us assume that there are $M^{o}$ channels in the sensing list for an IoT device *i*. Each channel *k* is described by L parameters. Let ω = $\omega_{1}$, $\omega_{2}$, …, $\omega_{L}$ be the weight vector and $\omega_{l}$ be the weight of *l*-th parameters. Let $I=[u_{k,l}]$ be the intermediate matrix that is obtained by multiplying channels with their parameters. To make the decision matrix, the intermediate matrix I is multiplied with weight vector ω (i.e., $B={\left[b_{k,l}\right]}_{M^{o}*L})$. The theoretical best and theoretically worse solution can be calculated as follows [50]:

(27) $$B^{+}=\left\{b_{1}^{+},b_{2}^{+},\ldots ,b_{M}^{o+}\right\},$$

(28) $$B^{-}=\left\{b_{1}^{-},b_{2}^{-},\ldots ,b_{M}^{o-}\right\},$$

where

(29) $$\begin{array}{c} b_{l}^{+}=max\left\{b_{k,l}|k=1,2,\ldots ,M^{o}\right\}\;\\ =\omega_{l}max\left\{k=1,2,\ldots ,M^{o}\right\}=\omega_{l}u_{l}^{+}, \end{array}$$

(30) $$\begin{array}{c} b_{l}^{-}=max\left\{b_{k,k}|k=1,2,\ldots ,M^{o}\right\}\;\\ =\omega_{l}min\left\{k=1,2,\ldots ,M^{o}\right\}=\omega_{l}u_{l}^{-}. \end{array}$$

For each channel, the distance vectors for ideal positve and ideal negative can be described using Equations (31) and (32) as follows [37]:

(31) $$\phi_{k}^{-}=\sum_{l=1}^{L}{\left(b_{k,l}-b_{l}^{+}\right)}^{2}=\sum_{l=1}^{L}{\left(\omega_{l}^{2}u_{k,l}-u_{l}^{+}\right)}^{2},$$

(32) $$\phi_{k}^{-}=\sum_{l=1}^{L}{\left(b_{k,l}-b_{l}^{-}\right)}^{2}=\sum_{l=1}^{L}{\left(\omega_{l}^{2}u_{k,l}-u_{l}^{-}\right)}^{2}.$$

The adjacency variable $\Psi_{l}$ can be computed as follows:

(33) $$\Psi_{k}=\phi_{k}^{-}/\left(\phi_{k}^{-}+\phi_{k}^{+}\right).$$

Using the adjacency variable $\Psi_{k}$, the optimization problem for channel ordering can be described as follows:

(34) $$\begin{array}{cc} \mathrm{Maximize} & \sum_{i=1}^{N}\sum_{k=1}^{M^{o}}\Psi_{k}^{+},\\ \mathrm{Subject}\;\mathrm{to} & \sum_{l=1}^{L}\omega_{l}=1\;\forall k,l. \end{array}$$

By maximizing adjacency function, we can obtain optimal CSO for each device *i*.

## 4. Performance Evaluation

This section provides the detailed simulation results and comparative analysis of proposed ODR, OSR and ORR schemes. The performance metrics selected for comparison are (1) PoD, (2) PoFA, (3) PoC, (4) data rate, and (5) average blocking and idle time probabilities. The comparisons are shown by varying the SNR(dB), 5G user activity, number of available channels, device density and frames, etc. The Monte Carlo principle is implemented to get average results over 500 different iterations. All results are acquired using Matlab 2017. Although the presented results are simulated for selected values, all results are equally applicable for any generic scenario with any number of IoT devices and available channels. We consider that microcells cover an area of an area of 1000 × 1000 m${}^{2}$ with $\bar{P}$ = 100 showing on–off activity across different time and space within the microcell. The STBS is transmitting with the power of 46 (dBm) and it is perfectly synchronized with IoT devices and PTBS. The PTBS is located at position ($X_{PTBS}=250,Y_{PTBS}=250)$ and STBS is operating at $\left(X_{STBS}=650,Y_{STBS}=650\right)$. For ODR, 5G users are randomly put in on and off states with probability 0.0∼0.60 and it is detected at IoT devices with total sensing duration of 10 μs. For ODR, we consider *L* = 2 with weights $\omega =[\omega_{1},\omega_{2}$] = [0.5, 0.5] where $\omega_{1}$ represents data rate and $\omega_{2}$ is the idle time probability of the channel. To highlight the effectiveness of the proposed scheme, we compare the performance of ODR with SCS [16] and OSR, ODR with DCSS [25] and DRCSS [26]. The rest of simulation parameters are shown in Table 3 for a better understanding of the simulation environment.

**Figure 5:**
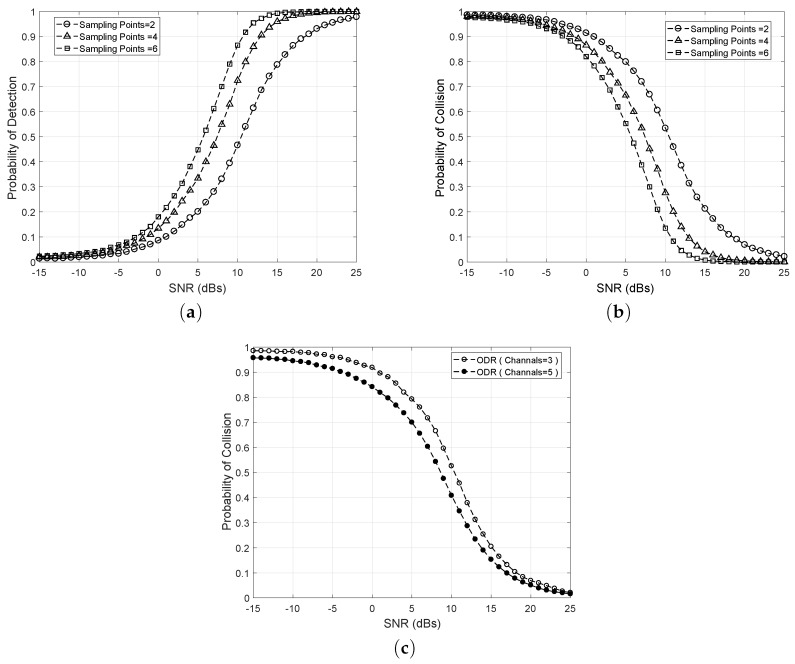
Performance gain of ODR (**a**) SNR vs. ${\bar{Q}}_{d,i,k};$ (**b**) SNR vs. ${\bar{Q}}_{c,i,k};$ (**c**) SNR vs. ${\bar{Q}}_{c,i,k}$ for $M^{o}=3$ and $M^{o}=5$.

Figure 5 illustrates the performance of ODR in terms of ${\bar{Q}}_{d,i,k}$ and ${\bar{Q}}_{c,i,k}$ across different SNR values between −15 dB ∼ 25 dB. The result depicts the higher performance gain of ODR at higher SNR values of the channel. Furthermore, the sensing samples (i.e., how many times the activity of the 5G user is detected) provide another insight into the sensitivity of the proposed scheme. For example, at SNR = 10 dB, the ${\bar{Q}}_{d,i,k}$ increases from 47% to 87% when sampling points are increased from 2 to 6 as shown in Figure 5a. Similarly, the probability of collision ${\bar{Q}}_{c,i,k}$ is presented in Figure 5b, which highlights the fact that collision probability decreases with the improvement in the SNR values of the detected channel and it decreases dramatically if we increase the sensing samples. For example, at SNR = 10 dB, the ${\bar{Q}}_{c,i,k}$ decreases from 55% to 13% as sampling points are increased from 2 to 6. Figure 5c explicitly illustrates the performance of ODR across multiple sensing channels. By sensing multichannel, the collision probability reduces, which increases the spectral efficiency and utilization. For example, at SNR = 10 dB, ${\bar{Q}}_{c,i,k}$ decreases from 53% to 40% when the number of channels are increased from $M^{o}$ = 3 to $M^{o}$ = 5.

**Figure 6:**
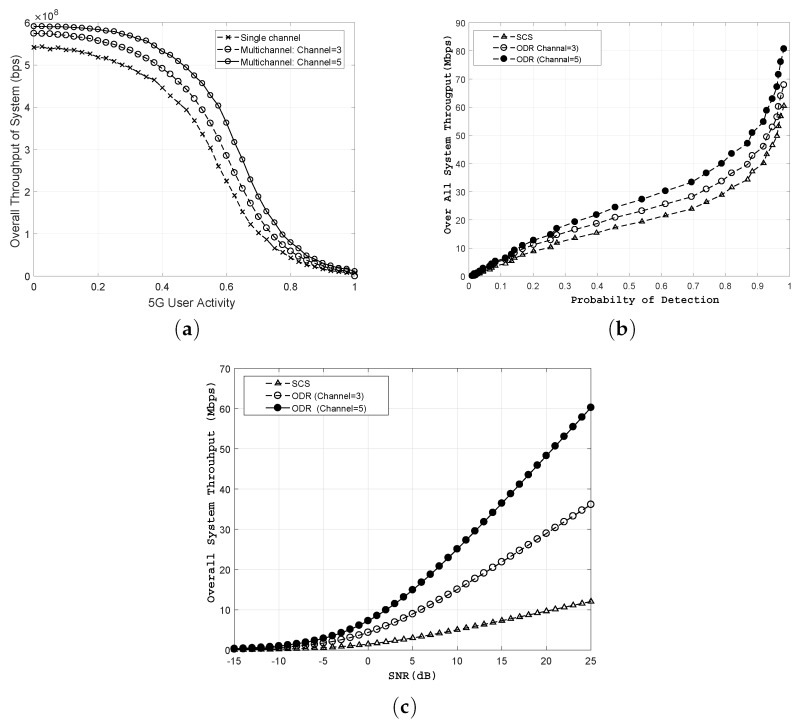
Performance gain of ODR (**a**) 5G user activity vs. system throughput; (**b**) ${\bar{Q}}_{d,i,k}$ vs. system throughput; (**c**) SNR vs. system throughput, for single ($M^{o}=1$), and multiple channels (i.e., $M^{0}=3$ and $M^{o}=5$).

Figure 6 presents the comparative analysis of the proposed ODR scheme with existing schemes in terms of throughput. For current simulation results, we consider N=50 with six sensing samples during a sensing duration $T_{sens}$. Two values of sensing channels (i.e., $M^{o}$ = 3 and $M^{o}$ = 5) are considered for comparison. The throughput is computed by allowing the IoT devices to transmit data on channels detected through ODR and scheduled using OSR. The result patterns demonstrate that the proposed ODR scheme provides more opportunities for spectrum utilization that will increase the overall throughput of the secondary tier network. The 5G user activity versus throughput is shown in Figure 6a. Results show that the throughput decreases when 5G user activity increases (i.e., $P_{busy}^{k}$ increases over available channels). Despite the decline in the throughput of IoT system with an increase in the 5G user activity, the proposed ODR scheme performs better than the existing single channel based spectrum sensing mechanisms. For example, at 5G user activity of $P_{busy}^{k}$ = 0.4, the ODR provides 9.6% with $M^{o}$ = 3 and 19.23% with $M^{o}$ = 5 better throughput compared to single channel-sensing schemes. Similarly, Figure 6b shows performance gain in terms of throughput across ${\bar{Q}}_{d,i,k}$. It can be seen from the results that ODR exhibits 22.6% and 45.04% better performance for cases of $M^{o}$ = 3 and $M^{o}$ = 5, respectively, compared to the SCS scheme at $P_{busy}^{k}$ = 0.7. Furthermore, the ODR shows significantly higher throughput compared to SCS for different SNR values of channels. For example, at 20 dB SNR, the ODR depicts three times and five times higher throughput gain compared to SCS.

**Figure 7:**
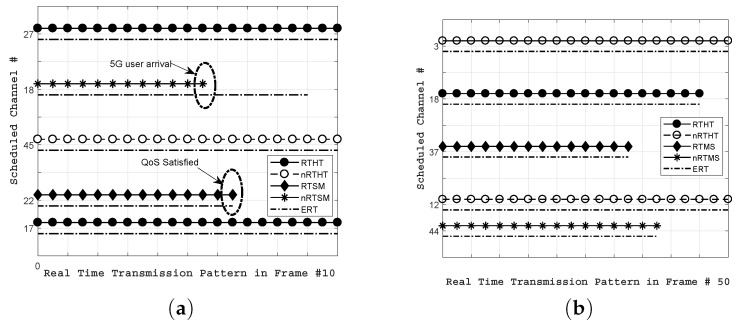
QoS support for IoT devices (**a**) *f*rame # 10; (**b**) *f*rame # 50.

Figure 7 exhibits the channel allocated to different IoT devices for two different numbers of frames using CMF. For the current result, we consider N = 50 IoT devices and *k* = 100 different channels with bandwidth $W_{k}$ varies from 5 KHz to 10 MHz. The traffic is generated using four classes depicted in Table 2 and simulation parameters highlighted in Table 3. Figure 7a depicts the scheduling pattern generated through OSR to IoT devices in frame *f* = 10. The result illustrates that proposed CMF allocates the best channels to meet the QoS requirements of heterogeneous IoT devices. The estimated required time (ERT) of an IoT device *i* is the minimal required time to complete the transmission, which is obtained by dividing the size of the data with the transmission time of frame *f*. For simplicity, we assume the same transmission time for all frames. There are two noticeable points in the result. First, the nRTSM traffic on channel #18 experiences interruption due to the arrival of the 5G user. The collision occurs here and we consider that the IoT device immediately halts its transmission and waits until the new channel is allocated to it. Second, considering the IoT device on channel #22, the RTMS traffic is quickly transmitted due to the availability of a higher quality and stable channel. Similarly, the scheduling pattern shown in Figure 7b for frame *f* = 50 supports the stated argument and highlights the fact that all users meet their QoS even earlier than the scheduled transmission time of frame *f*. Hence, the proposed CMF successfully meets the heterogeneous requirements of different kinds of IoT devices.

**Figure 8:**
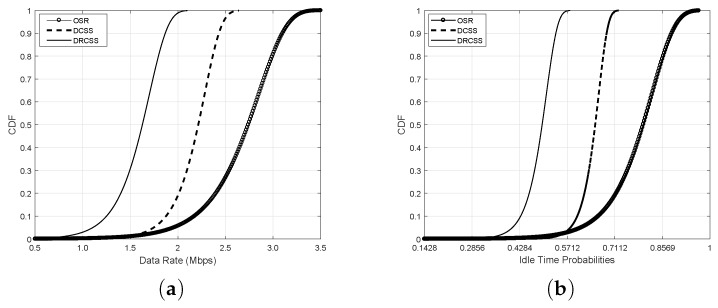
CDF based comparison with existing schemes (**a**) data rate (Mbps); (**b**) idle time probabilities.

To further extend the comparison, the cumulative distribution function (CDF) of data rate and idle time probabilities of proposed CMF are depicted in Figure 8 along with DCSS [25] and DRCSS [26]. The DCSS schedules the channels using a greedy approach, whereas the DRCSS employs a random channel scheduling mechanism. The proposed scheme outperforms the existing schemes for both factors and provides high quality (i.e., high data rate) and stable (i.e., higher idle time probabilities) channels to IoT for their transmission. For example, in Figure 8a, the CMF provides data rates between 1.0∼3.5 Mbps, whereas DCSS and DRCSS schedule channels with data rates between 0.85∼2.68 and 0.67∼2 Mbps, respectively. Hence, the OSR provides 25% and 75% higher data rates compared to DCSS and DRCSS, respectively. Similarly, the OSR provides 36.61% and 70% more stable channels compared to DCSS and DRCSS, respectively, as depicted in Figure 8b. This performance gain is achieved due to the robust operation of ODR and ORR, which helps to locate the best quality channels in terms of data rate and idle time probabilities.

**Figure 9:**
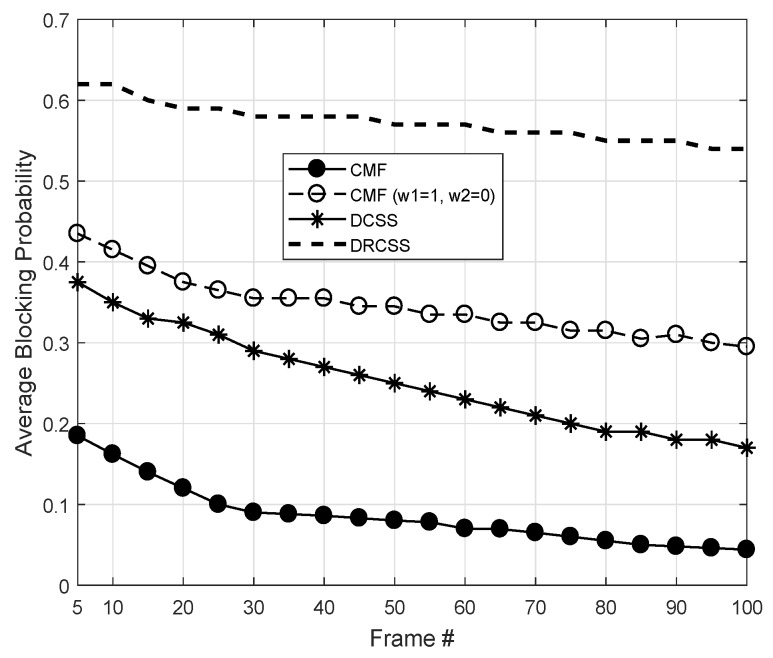
Average blocking probabilities of unified CMF and existing schemes.

**Figure 10:**
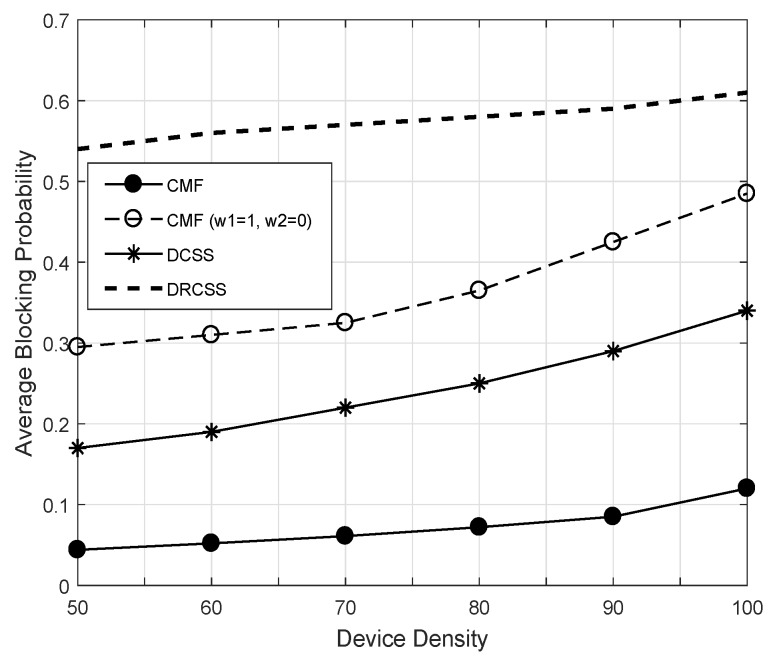
Average blocking probabilities of unified CMF and existing schemes.

Figure 9 illustrates the performance gain of CMF across different frame indexes for $M^{o}$ = 5. The results are plotted for two settings (i.e., ORR with Case 1: $\omega_{1}$ = 0.5 and $\omega_{2}$ = 0.5 and Case 2: $\omega_{1}$ = 1 and $\omega_{2}$ = 0) in comparison with DCSS and DRCSS. Initially, the CMF shows few higher blocking probabilities, but, with the ORR and ODR, it significantly reduces average blocking probability. The multichannel based ODR diversifies the opportunities and ORR through CSO directs IoT to sense only high throughput and higher idle probability channels. For example, at frame index 100, the CMF shows three times lower blocking probability compared to DCSS. The performance gain over DRCSS is even drastically higher. Similarly, Figure 10 shows the comparison across different device densities. Once again, the proposed CMF shows a significantly higher performance gain. Hence, from the obtained results, it is evident that the proposed CMF is quite effective in supporting the communication of IoT devices.

## 5. Conclusions

This paper introduces a novel channel management framework for the joint operation of IoT and 5G communication where IoT devices are equipped with cognitive radio technology to avail a wide range of non contiguous spectrum for the optimal operation. The unified CMF addresses three main concerns including opportunity detection, opportunity scheduling and opportunity ranking. For opportunity detection, the proposed ODR scheme maximizes spectrum utilization and provides up to three times higher throughput compared to the existing single channel sensing schemes. For opportunity scheduling, we employ an ILP based optimization scheme that provides channels to IoT devices for their transmission considering their individual QoS requirements in terms of data rate and stability. To further extend the capability of CMF, a novel opportunity ranking scheme is introduced that provides special CSO to IoT devices for ODR for frame *f*+1 by taking channel feedback about transmission experience during frame *f*. Results show that the proposed novel framework meets individual QoS requirements and provides up to three times higher throughput and stable channels compared to the existing schemes. In the future, this work can be extended to devise a scheme that can perform ORR and ODR using a single optimization problem. Furthermore, similar to ODR, the multichannel allocation can be done in OSR.

## Figures and Tables

**Table 1:** Symbols and notations.

Abbreviations & Symbols	Meaning
*ODR*	Opportunity detector
OSR	Opportunity Scheduler
ORR	Opportunity ranker
STBS	Secondary-tier base station
CMF	Channel management framework
PTU	Primary-tier user
STU	Secondary-tier user
S	Number of 5G users
N	Number of IoT devices
M	Number of channels
$M^{o}$	Channels to be sensed by an IoT device
*i*	Subscript of IoT devices
*k*	Subscript of channels
*f*	Subscript of frame index
*ERT*	Estimated required time for the transmission
*CSO*	Channel sensing order
*SCS*	Single channel sensing
*PTU*	Primary-tier user
$\lambda_{0}$	Single sensing threshold
*$\lambda_{1}$,$\lambda_{2}$*	Two thresholds for double threshold scheme
ω	weight vector used in ORR
$B^{+}$	Ideal positive solution in ORR
$B^{-}$	Ideal negative solution in ORR
$\zeta_{i}^{min}$	Minimum data rate requirement of an IoT device *i*
r(t)	Received signal at an IoT device
h(t)	Channel response
η(t)	Noise contents
*$P_{d}$,$P_{c}$,$P_{fa}$*	PoD, PoC and PoFA for non fading case with single channel, $M^{o}=1$
*$Q_{d}$,$Q_{c}$,$Q_{fa}$*	PoD, PoC and PoFA for fading case
*${\bar{Q}}_{d}$,${\bar{Q}}_{c}$,${\bar{Q}}_{fa}$*	PoD, PoC and PoFA for multichannel case $M^{o}>1$
$D_{i,f,k}^{est}$	Estimated data size of IoT device *i* to be sent on channel *k* during frame *f*
$X_{i,k}$	Binary decision variable for OSR
$\phi_{k}^{+}$	Distance vector for ideal positive solution
$\phi_{k}^{-}$	Distance vector for ideal negative solution

**Table 2:** QoS parameters for different IoT classes [7,30].

Traffic	IoT Minimum QoS Requirements	
	Data Rate (Kbps)	Idle Probability
	($\zeta^{\min}$)	($\rho^{\min}$)
RTSM	10	0.7
nRTSM	5	≥0.4
RTHT	90	0.7
nRTHT	50	≥0.4

**Table 3:** Simulation parameters.

Parameters	Values
Received SNR of IoT devices	−15∼25 dBm
Noise PSD	−115 dBm/Hz
Channels	10∼30
5G User activity	0.0∼0.6
Frame duration (*f*)	10 ms
Sensing duration	10 μs
Sampling frequency of sensing	100 kHz
Threshold ($\lambda_{o}$)	0∼40
Lower threshold ($\lambda_{1}$)	0.8$\lambda_{o}$
Upper threshold ($\lambda_{2}$)	1.2$\lambda_{o}$
Modulation scheme	MQAM
Weights [data rate $\omega_{1}$, 5G user activity $\omega_{2}$]	[0.5, 0.5]
